# Ultraviolet Light Generation through Lanthanide Upconversion

**DOI:** 10.1021/acs.accounts.5c00684

**Published:** 2025-12-29

**Authors:** Leipeng Li, Hao Suo, Feng Wang

**Affiliations:** † Department of Materials Science and Engineering, 53025 City University of Hong Kong, 83 Tat Chee Avenue, Hong Kong SAR, China; ‡ College of Physics Science and Technology, Hebei University, Baoding 071002, China; § Hong Kong Institute for Clean Energy, City University of Hong Kong, 83 Tat Chee Avenue, Hong Kong SAR, China

## Abstract

Upconversion is a nonlinear optical process in which long-wavelength photons are absorbed by specific material systems and converted into shorter-wavelength light. Yb^3+^–Ln^3+^ (Ln: Er/Ho/Tm) pairs are the most widely studied upconversion systems, demonstrating great success in efficient near-infrared-to-visible light conversion. Nevertheless, further exploration of upconversion luminescence toward shorter wavelengths, especially in the UV region, has achieved limited progress. In comparison with visible light, UV radiation suffers from minimized interference from natural and most artificial light sources. By shifting the emission to the deep UV band, for example, solar interference could be circumvented, enabling highly valuable applications such as solar-blind imaging and labeling. Additionally, due to the higher photon energy in this spectral range, the system could be simultaneously employed for sterilization, phototherapy, and plastic degradation.

To unlock the application potentials of UV-emitting upconversion materials, substantial research efforts have been undertaken in recent years. Specifically, classic visible upconverting Er^3+^ and Tm^3+^ ions have been repurposed for UV emission due to their rich energy levels extending to the UV spectrum region. To effectively populate the high-lying excited states, systematic investigations into doping concentrations, host lattice compositions, and excitation schemes have been conducted. In parallel, Pr^3+^—typically ineffective for near-infrared to visible upconversion—has been established as a prominent candidate for UV upconversion under blue-light excitation. By precisely tuning its 4f^1^5d^1^ state through host lattice engineering, both the upconversion dynamics and emission characteristics can be strategically optimized.

In this Account, we focus on recent advances in UV upconversion through lanthanide-doped inorganic crystals, primarily drawing upon our research group’s advancements over the past few years. We begin by summarizing the methods for constructing UV upconversion materials based on rational selection of dopant ions and host crystals, including Er^3+^-, Tm^3+^-, and Pr^3+^-based systems. Building on these foundations, we introduce emerging methods for enhancing the UV upconversion emission intensity, encompassing dielectric coupling, plasmonic modulation, and organic surface coating, which all have a certain degree of universality. The subsequent section will focus on the frontier applications of UV upconversion in lighting, imaging, and environmental sciences. In the end, we conclude by providing a summary and a perspective on future directions.

## Graphical abstract

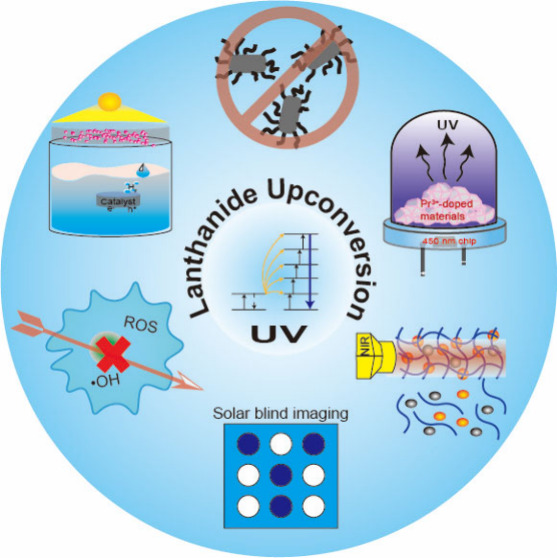

## Key References

- Suo, H.; Zhao, P.; Zhang, X.; Guo, Y.; Guo, D.; Chang, J.; Chen, J.; Li, P.; Wang, Z.; Wei, H.; Zheng, W.; Wang, F. Bright Upconversion over Extended Temperatures Enabled by an Organic Surface Layer. *Nat. Commun*. **2025**, *16*, 3249.^1^ This work proposed a versatile strategy to boost upconversion efficiency, including the UV emission (345 nm: ^1^I_6_ → ^3^F_4_ of Tm^3+^) of NaGdF_4_:Yb^3+^/Tm^3+^, across a wide temperature range by surface coordination of small organic molecules.
- Du, Y.; Jin, Z.; Li, Z.; Sun, T.; Meng, H.; Jiang, X.; Wang, Y.; Peng, D.; Li, J.; Wang, A.; Zou, H.; Rao, F.; Wang, F.; Chen, X. Tuning the 5d State of Pr^3+^ in Oxyhalides for Efficient Deep Ultraviolet Upconversion. *Adv. Optical Mater*. **2024**, *12*, 2400971.^2^ This work introduced a novel class of Pr^3+^-doped rare-earth oxyhalides (YOCl, YOBr, and LuOBr) to achieve efficient upconverted deep UV emission from the 5d → 4f transition of Pr^3+^ in the spectrum range of 250–350 nm.
- Sun, T.; Chen, B.; Guo, Y.; Zhu, Q.; Zhao, J.; Li, Y.; Chen, X.; Wu, Y.; Gao, Y.; Jin, L.; Chu, S. T.; Wang, F. Ultralarge anti-Stokes Lasing through Tandem Upconversion. *Nat. Commun*. **2022**, *13*, 1032.^3^ This work designed a core–shell–shell nanoparticle capable of converting 1550 nm photons into 290 nm light (^1^I_6_ → ^3^H_6_ of Tm^3+^), showing great potential for miniaturized single-mode UV lasing at 289.2 nm.
- Sun, T.; Li, Y.; Ho, W. L.; Zhu, Q.; Chen, X.; Jin, L.; Zhu, H.; Huang, B.; Lin, J.; Little, B. E.; Chu, S. T.; Wang, F. Integrating Temporal and Spatial Control of Electronic Transitions for Bright Multiphoton Upconversion. *Nat. Commun*. **2019**, *10*, 1811.^4^ This work proposed a general strategy for boosting the upconversion efficiency of Er^3+^ emission by combining a core–shell nanostructured host and an integrated optical waveguide circuit excitation platform.

## 1 Introduction

Upconversion is a nonlinear optical process in which low-energy photons are absorbed by specific material systems and converted into higher-energy light emission.^5,6^ The emitted photons exhibit an unusual spectral shift relative to the absorbed light, known as the anti-Stokes shift, which can sometimes be exceptionally large, reaching even thousands of nanometers.^2^ Such anti-Stokes shift property endows upconversion luminescence with broad application prospects in biodetection and sensing, information storage, and anticounterfeiting, some of which have already transitioned from laboratory research to real-world applications. Selective lanthanide ions, alone or in combination, can be embedded in inorganic crystal lattices and excited by near-infrared (NIR) lasers to achieve tunable upconversion emissions according to their unique energy-level structures.^7^ Taking the Yb^3+^/Er^3+^ codoped system as an example, Yb^3+^ ions efficiently absorb 980 nm photons and subsequently transfer the energy to Er^3+^ ions, resulting in upconversion luminescence from the latter. Similar to the Yb^3+^/Er^3+^ pair, Yb^3+^/Ho^3+^, Yb^3+^/Tm^3+^, and Yb^3+^/Nd^3+^ are also widely studied upconversion systems. In these combinations, the primary research focus lies in achieving efficient NIR-to-visible light conversion, including red, green, yellow, or blue-violet emissions.^7–10^ These visible upconversion emissions enable diverse applications, such as remote deep brain modulation for therapeutic dissection of Parkinson’s disease and thermal-quenching-resistant temperature sensing, among others.^11–14^

Given that the visible upconversion emissions substantially overlap with common light sources in daily life, particularly white light sources, their application may be compromised in certain scenarios. For instance, utilizing the intensity ratio of Er^3+^ green emissions for temperature sensing under indoor lighting conditions suffers from severe interference from white light-emitting diode (LED) light.^15^ A feasible solution is to further explore the potential of upconversion luminescence in the shorter-wavelength regions, ideally extending into the UV region.^16–19^ If the emission can be shifted to the ultraviolet B (UVB, 280–315 nm) or even ultraviolet C (UVC, 100–280 nm) band, solar interference could also be circumvented, enabling highly valuable applications such as solar-blind imaging and labeling.^20–24^ It is important to note that the UVC band is often referred to as the 200–280 nm range in practice due to atmospheric absorption and measurement difficulty below 200 nm. Additionally, due to the higher photon energy in this spectral range, the system could be simultaneously employed for an array of photon-activation processes, including disinfection, degradation of polymers, and photocatalytic water splitting (Figure 1).^2,3,25–28^

**Figure 1:**
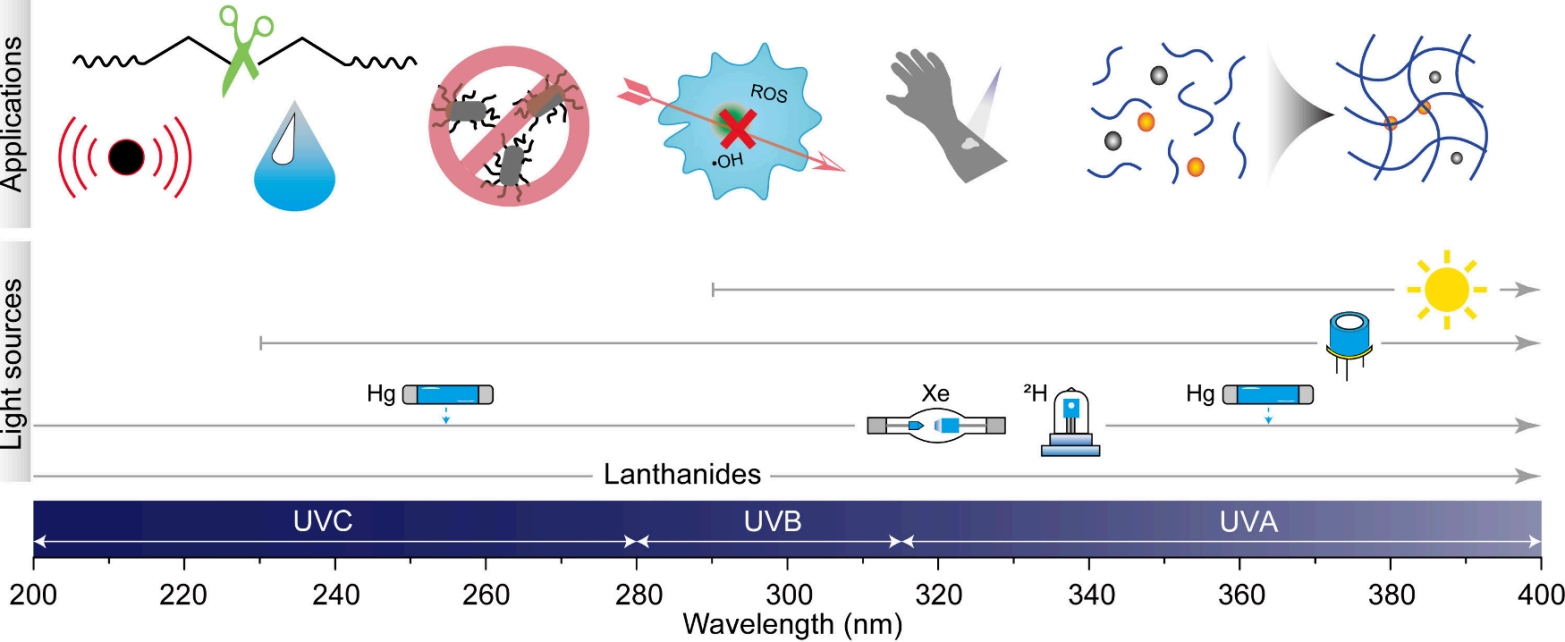
Summary of light sources capable of emitting photons within the 200–400 nm range, as well as the potential applications.

In this Account, we focus on recent progress in constructing and utilizing UV upconversion luminescence, primarily drawing upon our research group’s advancements over the past few years. First, we survey recently developed materials systems incorporated with lanthanide dopants for performing UV upconversion. We also discuss various strategies, such as surface coating with organic ligands, for enhancing the upconversion processes in these materials. Subsequently, we highlight exploratory applications of UV upconversion luminescence, such as lasing and lighting, solar blind imaging, UV-light-initiated disinfection, etc. Finally, we address existing challenges in the field and propose potential solutions.

## 2 METHODS FOR ACHIEVING UV UPCONVERSION

Several lanthanide ions, including Er^3+^, Ho^3+^, Tm^3+^, Nd^3+^, and Pr^3+^, have been reported to exhibit UV upconversion luminescence due to their rich energy level structure extending into the UV region.^29–31^ For instance, the emissions of Nd^3+^ following 577 nm excitation can reach the UVA band, with example transitions including ^4^D_3/2_ → ^4^I_9/2_ (354 nm) and ^4^D_3/2_ → ^4^I_11/2_/^2^P_3/2_ → ^4^I_9/2_ (382 nm).^30^ However, a common limitation for many of these UV transitions is their inherently low intensity, even at extremely high excitation power densities. Current studies mainly employ Er^3+^, Tm^3+^, and Pr^3+^ as activators for UV upconversion.

### 2.1 Er^3+^-Based Systems

Er^3+^ typically exhibits UV emission peaks at ≈380 nm (^4^G_11/2_ → ^4^I_15/2_) and ≈315 nm (^2^P_3/2_ → ^4^I_15/2_) (Figure 2a).^32^ Although shorter-wavelength transitions within the UVB and UVC bands, such as ^2^K_13/2_ → ^4^I_15/2_ (304 nm) and ^4^D_7/2_ → ^4^I_15/2_ (255 nm), are known, their intensities are generally too low to be detected in most host matrices.^33^ Therefore, this section mainly involves the 380 nm (^4^G_11/2_ → ^4^I_15/2_) and 315 nm (^2^P_3/2_ → ^4^I_15/2_) emission lines of Er^3+^ through an upconversion route.

**Figure 2:**
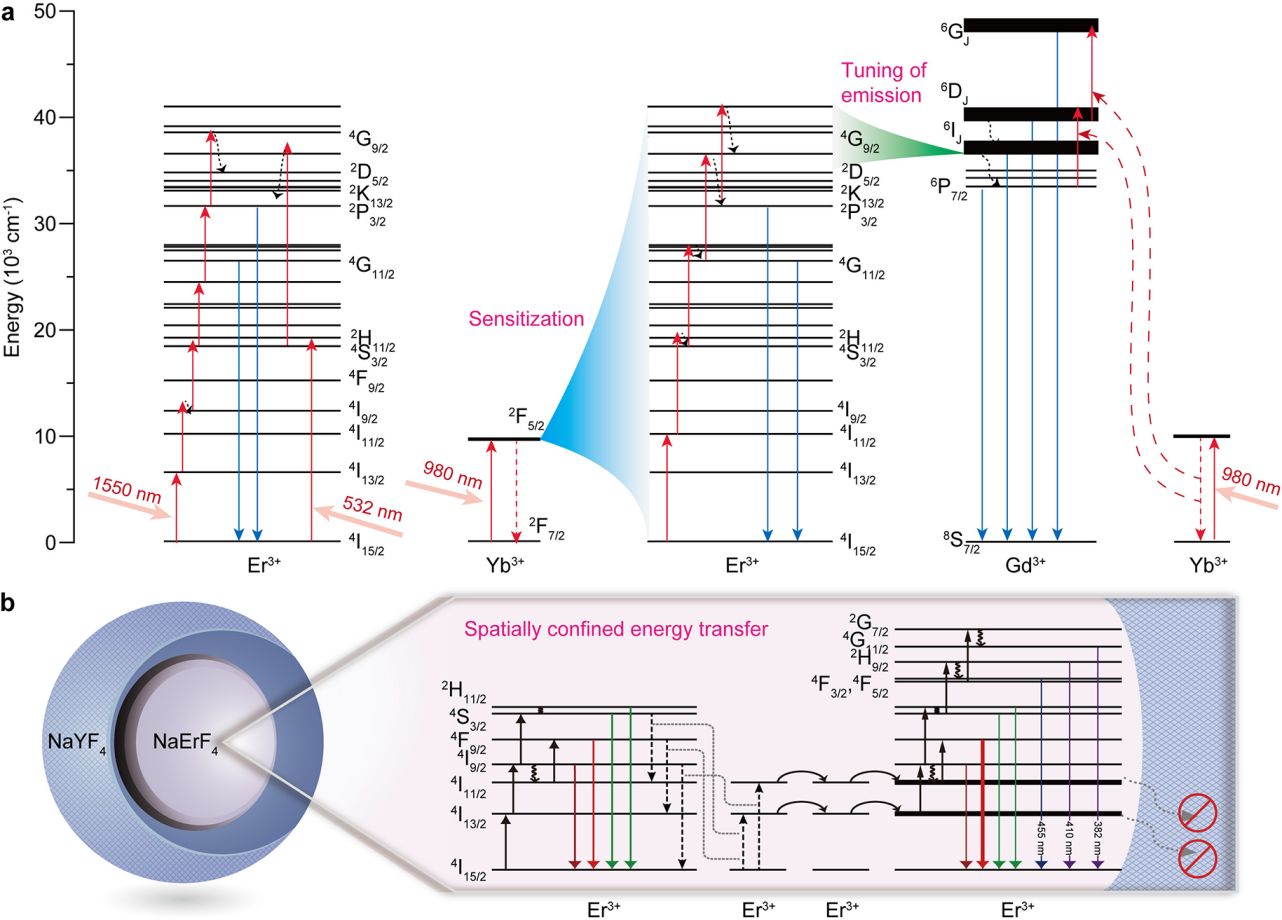
(a) Schematic diagram of Er^3+^-based systems for generating UV upconversion emission. (b) Mitigation of concentration quenching in NaErF_4_@NaYF_4_ nanoparticles under 1532 nm excitation. Adapted with permission from ref 4. Copyright 2019 Springer Nature.

Er^3+^ alone can achieve UV upconversion luminescence by different excitation schemes. A 532 nm laser serves as a suitable excitation source for Er^3+^ in various hosts such as BaGd_2_ZnO_5_,^34^ as its photon energy matches the energy gap between the ^4^I_15/2_ ground state and the ^2^H_11/2_/^4^S_3/2_ excited states of Er^3+^ ions. After population of the ^2^H_11/2_/^4^S_3/2_ states is established by 532 nm excitation, subsequent transitions to even higher-lying energy levels may occur via excited-state absorption (ESA) or energy transfer upconversion (ETU), resulting in UV upconversion emission with wavelengths as short as ≈200 nm. Alternatively, UV upconversion emission covering the 250–400 nm range, with the shortest wavelength peak at 276 nm, can be obtained following 1550 nm laser excitation into the ^4^I_15/2_ → ^4^I_13/2_ transition and a series of ESA and/or ETU processes. The six-photon upconversion, as demonstrated in SiAlON:Er^3+^ ceramics, is largely enabled by a relatively efficient photoexcitation process.^35^

Yb^3+^ ion with a large absorption cross-section at 980 nm (∼10^–20^ cm^2^) is frequently used as a sensitizer to promote the UV upconversion luminescence of Er^3+^ (Figure 2a). In Yb^3+^/Er^3+^ codoped NaGdF_4_, Gd^3+^ can expand the UV emission spectrum.^33^ This phenomenon is attributed to an energy transfer (ET) process from high-energy excited states (e.g., ^4^D_5/2_, ^4^G_7/2_, and ^2^K_13/2_) of Er^3+^ to Gd^3+^, inducing the most prominent Gd^3+^ emission centered ≈310 nm, originating from the ^6^P_7/2_ → ^8^S_7/2_ transition. Furthermore, the Gd^3+^ ions in the ^6^P_7/2_ excited state can be further promoted to the ^6^D_J_ and ^6^G_J_ states by Yb^3+^, subsequently generating shorter-wavelength emissions.

Upconversion luminescence is strongly affected by the doping concentration of lanthanide ions. Conventionally, the doping concentration is generally limited to several mole percent to prevent detrimental cross-relaxation and long-distance energy migration, which may lead to concentration quenching of the luminescence. Recent investigations reveal that core–shell nanostructured host materials can effectively regulate ET interactions among dopant ions to mitigate concentration quenching.^36^ Consequently, unusually high concentrations of dopant ions can be employed to enhance UV upconversion emissions, stemming from increased optical carriers as well as enhanced interionic interaction that triggers efficient ET upconversion (Figure 2b). Specifically, we demonstrated a NaErF_4_@NaYF_4_ core–shell nanoparticle comprising 100% Er^3+^ in the core level, which exhibited a strong UVA band at 382 nm and a weak UVB line at 313 nm by 1550 nm excitation through Er^3+^ self-sensitized upconversion.^4^

### 2.2 Tm^3+^-Based Systems

UV upconversion in Tm^3+^ originates from the ^1^D_2_ and ^1^I_6_ excited states that give rise to UVB and UVA emissions at 290 nm (^1^I_6_ → ^3^H_6_), 345 nm (^1^I_6_ → ^3^F_4_), and 365 nm (^1^D_2_ → ^3^H_6_). Notably, other transitions like ^3^P_2_ → ^3^H_6_ (264 nm) have also been observed,^37^ but only under a high excitation power density or in very few matrices.

To facilitate the UV upconversion of Tm^3+^, Yb^3+^ is always employed as a sensitizer. Through Yb^3+^-to-Tm^3+^ ET, the energy difference in successive excitation steps can be compensated by phonons (Figure 3a). To maximize the sensitization effect, recent studies mostly adopt core–shell nanostructured host materials coupled with a high doping concentration of Yb^3+^. Owing to enhanced absorption of excitation light and strengthened ET to Tm^3+^ activators, continuously increasing Yb^3+^ doping concentrations substantially enhance populations of the high-energy states (^1^D_2_ and ^1^I_6_) responsible for UV upconversion.^38^ The efficient high-order upconversion in Tm^3+^ also enables ET to Gd^3+^ ions as supplementary dopants, resulting in UV emission at 310 nm through the ^6^P_7/2_ → ^8^S_7/2_ transition of Gd^3+^ in NaYF_4_@NaYbF_4_:Tm/Gd@NaYF_4_.

**Figure 3:**
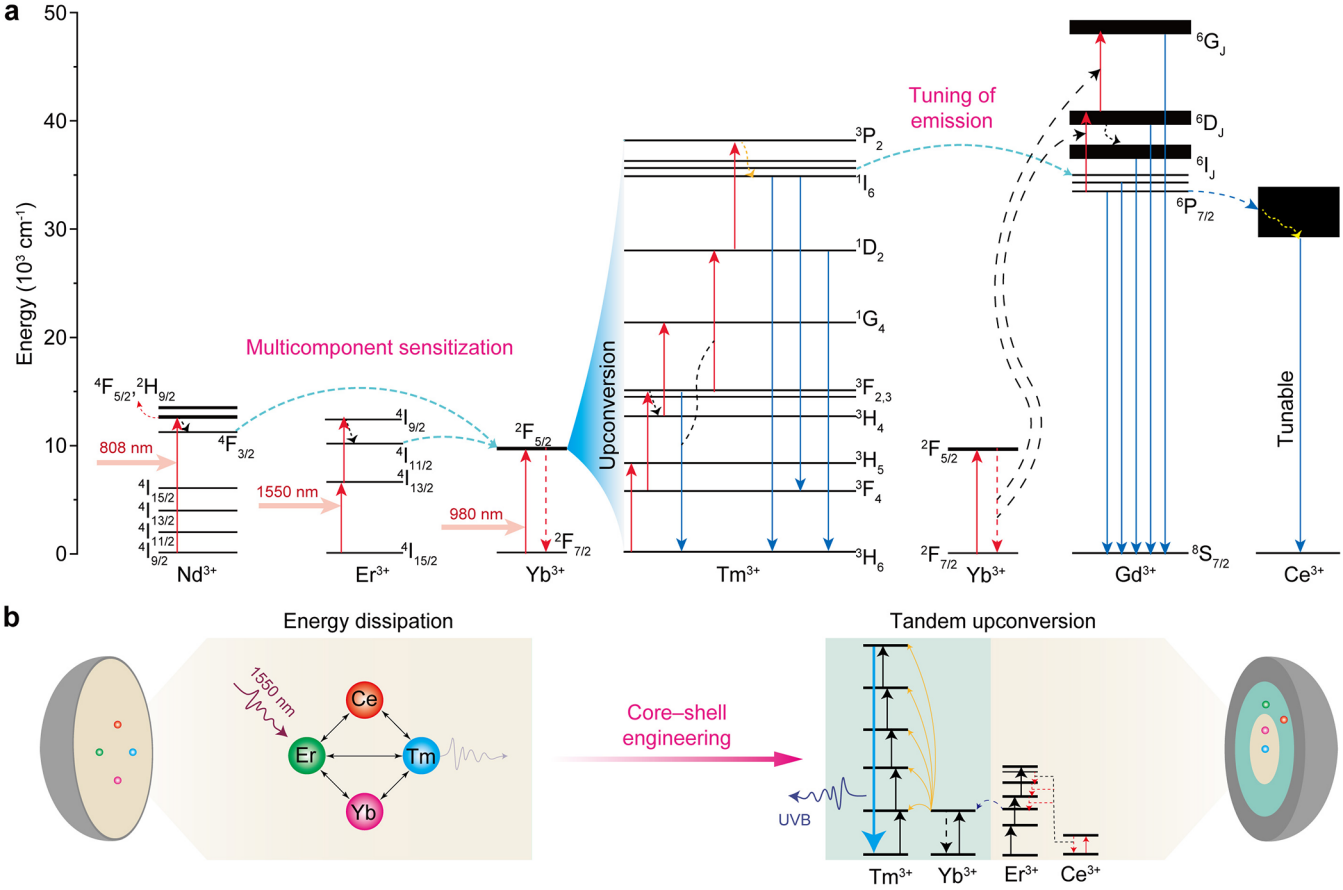
(a) Schematic diagram of Tm^3+^-based systems for generating UV upconversion emission. (b) Core–shell design for expanding the excitation spectrum of Tm^3+^ in NaYbF_4_:Tm/Gd@NaErF_4_:Ce@NaYF_4_.

By adopting active core/active shell nanostructures, otherwise incompatible dopant ions can be integrated into a single nanoparticle to substantially tune the emission and excitation spectra of Tm^3+^-based upconversion through interlayer ET. For example, using a NaYbF_4_:Gd/Tm@NaGdF_4_@CaF_2_:Ce nanostructure, we realized broadband UV emission of Ce^3+^ through Yb^3+^ → Tm^3+^ → Gd^3+^ → Ce^3+^ ET.^39^ Based on a similar strategy, we achieved UV upconversion of Tm^3+^ at 290 nm by 1550 nm excitation in a NaYF_4_:Yb/Tm@NaErF_4_:Ce@NaYF_4_ architecture through tandem upconversion, in which Er^3+^ self-sensitized upconversion in the inner shell layer triggers Yb^3+^-sensitized upconversion of Tm^3+^ in the core level (Figure 3b).^3^ In another example, Su et al. designed a NaGdF_4_:Yb/Tm@NaYF_4_:Yb@NaGdF_4_:Yb/Nd@NaGdF_4_ nanostructure.^40^ By confining sensitizer (Nd^3+^), migrator (Nd^3+^), emitter (Tm^3+^), and recycler (Gd^3+^) in different shells, six-photon-upconverted UV emission at 253 nm (Gd^3+^: ^6^D_J_ → ^8^S_7/2_ transition) was observed under 808 nm excitation. Notably, 808 nm excitation can circumvent the problem of overheating associated with absorption of 980 nm light by water molecules in biomedical applications.

### 2.3 Pr^3+^-Based Systems

In general, the UV luminescence of Pr^3+^ stems from the 4f^1^5d^1^ and ^1^S_0_ (4f^2^) excited states. However, the population of the ^1^S_0_ state by an upconversion process is infeasible due to the considerable energy gap to the next lower-lying state (≈23000 cm^–1^). Therefore, UV upconversion in Pr^3+^ ions is exclusively realized by the interconfigurational 5d → 4f transition, which features a broad bandwidth and tunable emission wavelength dependent on the host composition and structure (Figure 4a). Partly owing to the involvement of parity-allowed 4f → 5d transition, Pr^3+^ upconversion can be activated by blue LEDs or even sunlight, in contrast to Er^3+^-, Ho^3+^-, and Tm^3+^-based upconversion systems that are heavily reliant on high-intensity laser excitations.

**Figure 4:**
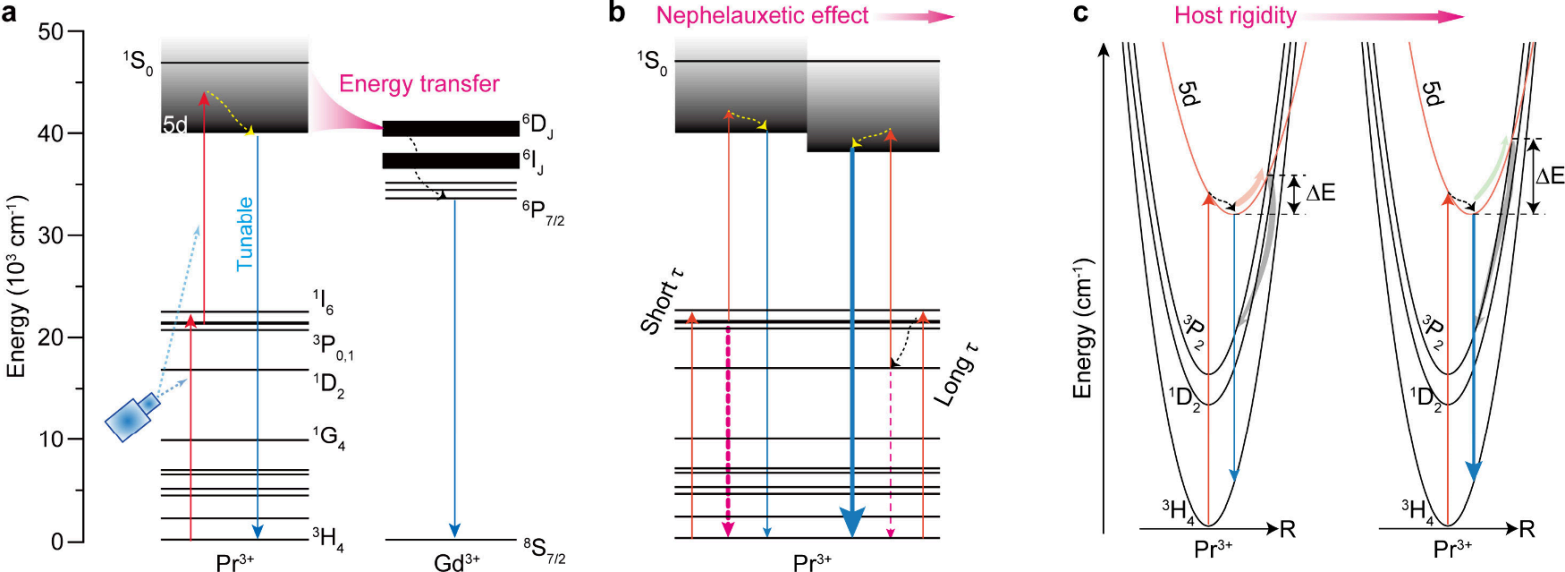
(a) Energy level diagrams of Pr^3+^ and Gd^3+^ showing the routes for the emissions of Pr^3+^ and Gd^3+^. (b) Rational utilization of long-lived ^1^D_2_ state for enhancing the UV upconversion luminescence of Pr^3+^. (c) Selection of rigid host lattices to prevent quenching of the 4f^1^5d^1^ state of Pr^3+^.

Due to the lack of suitable sensitizers, upconversion processes in Pr^3+^-based systems are typically dominated by ESA. In this connection, the selection of host materials is critical to place the 5d state at appropriate energy levels for the upconversion to proceed. Our investigations suggest that host materials exhibiting moderate covalency in their chemical bonds are favorable for facilitating the UV upconversion of Pr^3+^.^41^ Specifically, we observed a UVB upconversion emission line at 315 nm from Lu_6_O_5_F_8_:Pr/Gd@Lu_6_O_5_F_8_ under excitation at 450 nm, ascribed to the ^6^P_7/2_ → ^8^S_7/2_ transition of Gd^3+^ that extracted the upconverted energy of Pr^3+^ ions (Figure 4a).^41^ Note that the incorporation of Gd^3+^, characterized by a considerable energy gap of approximately 32,200 cm^–1^, is beneficial for preserving the excitation energy against multiphonon relaxation losses, in accordance with the energy gap law.^42^ The Pr^3+^ → Gd^3+^ ET following blue-light-excited upconversion was also observed in other systems such as Ba_2_SiO_4_:Pr/Gd and Y_7_O_6_F_9_:Pr/Gd.^43,44^

By virtue of the adjustable 4f^1^5d^1^ excited state, the excitation pathway can be rationally designed to optimize the upconversion performance. We leveraged this effect to enhance Pr^3+^ UV upconversion by using the long-lived ^1^D_2_ state (43.5 μs) as the intermediate state instead of the conventionally used ^3^P_0_ state with a relatively short lifetime (6.56 μs).^2^ This was achieved by replacing Cl with Br and Y with Lu in YOCl to lower down the 5d state of Pr^3+^ by leveraging the nephelauxetic effect (Figure 4b). As a result, we established excitation through ^3^H_4_ → ^1^D_2_ (via ^3^P_2,1,0_/^1^I_6_) → 4f^1^5d^1^ in LuOBr:Pr^3+^ by excitation of an Xe lamp at 450 nm, demonstrating 1.8- and 56.7-fold enhancement of UV upconversion emission than YOCl:Pr^3+^ and the benchmark Lu_7_O_6_F_9_:Pr^3+^, respectively. Derén et al. also highlighted the importance of utilizing the long-lived ^1^D_2_ over the short-lived ^3^P_J_ as the intermediate state, taking Sr_3_(BO_3_)_2_:Pr^3+^ as an example.^45^

The 4f^1^5d^1^ state of Pr^3+^ is subjected to quenching by crossover due to the offset of the configurational coordinate diagram relative to the lower-lying 4f states. In this regard, using a stiff crystal lattice is crucial for preserving the UV upconversion luminescence (Figure 4c).^2^ Our Crystal Orbital Hamilton Population (COHP) analysis shows that stronger covalent RE–O and RE–X bonds are formed with smaller cations (e.g., Lu^3+^) and larger anions (e.g., Br^–^).^2^ This bonding creates a rigid lattice with a small Stokes shift and offset, which in turn establishes a large energy barrier (Δ*E*) for crossover, thereby effectively inhibiting the quenching process.

## 3 STRATEGIES FOR BOOSTING UV UPCONVERSION

Lanthanide-doped upconversion materials generally suffer from low photon conversion efficiency, primarily due to the small absorption cross-section of lanthanide ions (∼10^–21^ cm^2^). Therefore, several strategies have been proposed to enhance the UV upconversion emission intensity by leveraging extrinsic factors such as dielectric microcavities, plasmonic nanostructures, and organic surface coatings.

To promote absorption of excitation light by lanthanide ions, we proposed an integrated optical waveguide circuit excitation platform (Figure 5a). By coupling the excitation light into a microring resonator, its power density can be amplified by several orders of magnitude as it circulates in the ring structure, providing efficient excitations to various upconversion materials situated on the resonator surface. In a case study, we achieved an increase of energy conversion efficiency from 1.1 to 5.0% for NaErF_4_@NaYF_4_ nanoparticles due to a substantially enhanced excitation process.^4^ In another investigation, we discovered that microcavity integration can suppress intrinsic recombination processes of intermediate excited states in embedded upconversion nanoparticles (UCNPs). As a result, the surplus of excitation power maximally populates the higher-lying excited states, which is in favor of multiphoton upconversion. By leveraging this effect, we achieved an order-of-magnitude enhancement for the 345 and 361 nm emissions of Tm^3+^ from NaYF_4_:Yb/Tm@NaYF_4_.^46^

**Figure 5:**
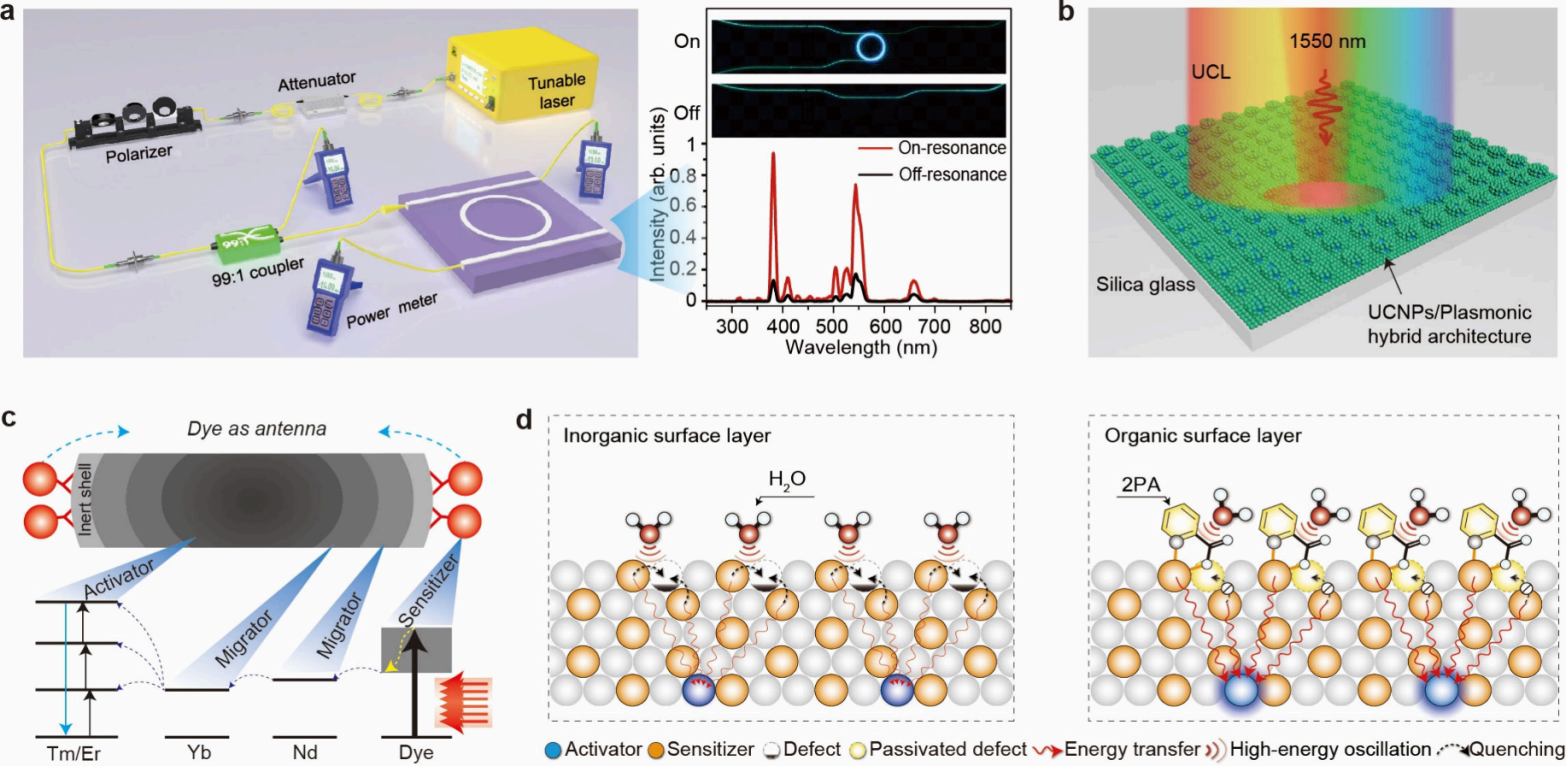
Schematic illustration of tuning/boosting UV upconversion through: (a) Fabrication of microcavity. Adapted with permission from ref 4. Copyright 2019 Springer Nature. (b) Surface plasmon resonance. Adapted with permission from ref 51. Copyright 2023 American Chemical Society. (c) Dye-sensitization and (d) organic surface layer coating. 2PA: bidentate picolinic acid. Adapted with permission from ref 1. Copyright 2025 Springer Nature.

Plasmonic modulation is a complex yet highly efficient strategy for enhancing upconversion luminescence (Figure 5b).^47–50^ The fundamental principle of this approach is to harness the surface plasmon resonance (SPR) of metallic nanostructures to enhance the luminescence of UCNPs. Specifically, by engineering the nanostructures to have a plasmon resonance that spectrally overlaps with the excitation wavelength, a greatly amplified local electric field is generated. This enhanced field boosts the excitation rate of the UCNPs, leading to a significant increase in upconversion emission. For instance, based on a UCNP–aluminum coupled system, Gao et al. reported the first-ever five-photon upconversion enhancement, from 1550 nm NIR excitation to 382 nm UV emission, achieving an 800-fold increase in intensity compared to the reference system.^51^

Another approach to address the light absorption issue is dye sensitization by leveraging the dye’s exceptionally large absorption cross-section (10^–17^∼10^–16^ cm^2^) and broad spectral response (Figure 5c).^52^ Chen et al. demonstrated a 25-fold enhancement for the upconversion emission at 350 nm of NaYbF_4_:Tm@NaYF_4_:Nd@IR-808-dye compared to that without dye sensitization.^53^ Similarly, Su’s group reported enhancements exceeding 600-fold in the 240–280 nm region, 300-fold in the 280–320 nm region, and 150-fold in the 320–400 nm region after coating IR-806 dye onto the surface of NaGdF_4_:Yb/Tm@NaYF_4_:Yb@NaGdF_4_:Yb/Nd@NaGdF_4_.^54^

Dye sensitization typically works for small UCNPs with dopant ions exposed to the nanoparticle surface for effective energy coupling with dye molecules. These nanoparticles usually contain massive surface defects that significantly quench the excitation energy. Although an inert epitaxial shell can passivate surface defects, it simultaneously increases the spatial separation between lanthanide ions and dye molecules, thereby diminishing the sensitization efficiency. Recently, our group discovered that bidentate picolinic acids (2PA) as a surface coating layer can effectively passivate surface defects without increasing the nanoparticle size (Figure 5d).^1^ These finding suggests that more effective dye sensitization might be achieved by combined use of fluorescent dyes with 2PA, or by designing dye molecules that incorporate the structural feature of 2PA.

## 4 UV UPCONVERSION FOR ADVANCED APPLICATIONS

A primary and straightforward application of UV upconversion involves fabricating light sources by embedding upconversion phosphors within appropriate matrices or micro/nanostructures, with prominent examples being lasers and LEDs. Moreover, the high energy inherent to UV photons allows for various specialized applications, such as disinfection, degradation of polymers, photocatalytic water splitting, etc. In addition, solar radiation in the UVB/C bands is effectively blocked by the atmospheric ozone layer, resulting in a ‘solar-blind’ condition at the Earth’s surface. Consequently, UV upconversion is highly advantageous for positioning and tracking, high contrast imaging, and anticounterfeiting.

### 4.1 Lasers and LEDs

Microlasers have wide applications in the fields of photonics and optoelectronics. Lanthanide-doped crystalline structures exhibiting highly tunable excitation and emission properties have emerged as promising microlasing materials, effectively extending the lasing wavelength into the UV range. Lanthanide-doped UCNPs are compatible with various solvents and exhibit excellent processability, allowing easy incorporation into polymers to fabricate microcavities. We constructed a simple bottle-shaped microcavity that supports whispering-gallery modes by coating an optical fiber with a nanoparticle–silica resin mixture (Figure 6a).^38^ This platform enabled us to demonstrate 311 nm lasing from Gd^3+^ (^6^P_7/2_ → ^8^S_7/2_ transition) through two different pathways: (i) ET from high-energy excited states of Tm^3+^ in NaYF_4_@NaYbF_4_:Tm/Gd@NaYF_4_ nanoparticles by NIR pumping,^38^ and (ii) ET from high-energy excited states of Pr^3+^ in Lu_6_O_5_F_8_:Pr/Gd@Lu_6_O_5_F_8_ nanoparticles by blue-light pumping.^41^ Moreover, the microcavity allows for straightforward tuning of key parameters, including mode spacing and threshold pump power, by adjusting its diameter. In 2022, our group demonstrated an alternative approach to achieve UVB lasing from the ^1^I_6_ → ^3^H_6_ transition of Tm^3+^ in NaYF_4_:Yb/Tm@NaErF_4_:Ce@NaYF_4_ nanoparticles (Figure 6b).^3^ Following sol–gel processing, the nanoparticles were incorporated into a toroidal silica microresonator as the laser cavity, which supports whispering gallery mode at the internal boundaries of the nanoparticle-doped microtoroidal resonator. Upon excitation at 1550 nm, the microresonator exhibited UVB lasing emission through Er^3+^-sensitized tandem upconversion.

**Figure 6:**
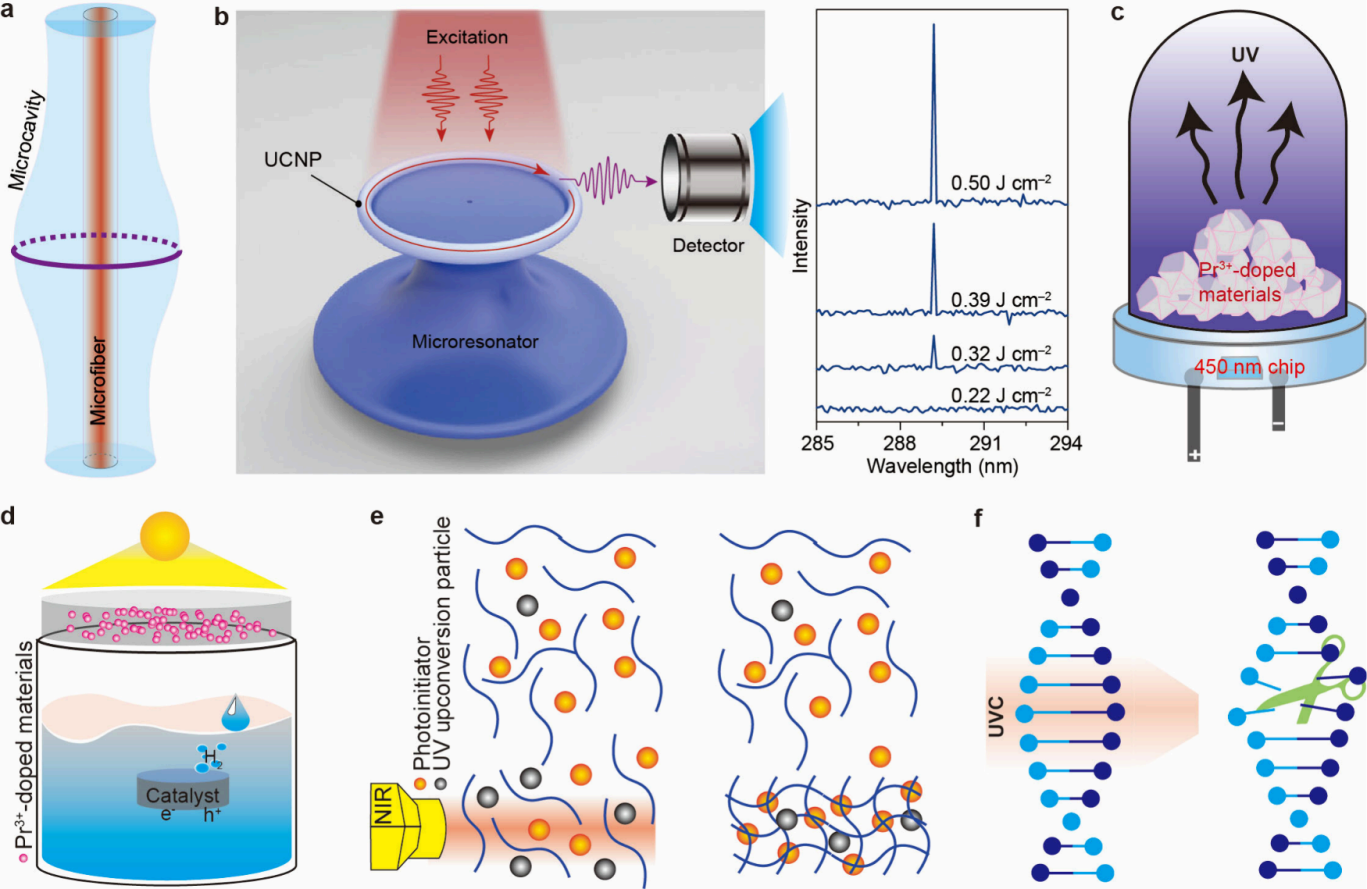
(a) Schematic diagram of a microcavity by coating the mixture of upconversion nanoparticles and silica resin on the surface of a bare optical fiber. (b) Schematic setup of the microtoroidal resonator platform containing NaYF_4_:Yb/Tm@NaErF_4_:Ce@NaYF_4_ for upconversion lasing. Adapted with permission from ref 3. Copyright 2022 Springer Nature. (c) Schematic diagram of a UV upconversion LED by coating Pr^3+^-doped materials on a 450 nm LED chip. Schematic diagram of high-energy related applications of UV upconversion: (d) water splitting, (e) photopolymerization, and (f) inactivating bacteria.

Song and co-workers reported a new method for cost-effective fabrication of on-chip integrated UVB microlasers.^55^ By spin-coating a LiYbF_4_:Tm@LiYbF_4_@LiLuF_4_ solution onto a patterned SiO_2_ substrate, high-quality lanthanide-based microdisks were formed by self-assembly on each SiO_2_ pillar. Relying on these thickness-adjustable microdisks, UVB whispering-gallery mode lasers at 289 nm (Tm^3+^: ^1^I_6_ → ^3^H_6_) were realized. The fabricated microdisks show excellent repeatability and are thus suitable for mass production. On this basis, they demonstrated switchable single-mode lasing with the emitting wavelength spanning up to 300 nm by integrating the reversely designed nanocrystals with two coupled microcavities of mismatched sizes.^56^ The same group also constructed a Fabry–Pérot laser with NaYbF_4_:Er@NaYbF_4_:Gd/Tm@NaGdF_4_@CaF_2_:Ce/epoxy composites as the gain medium.^57^ By taking advantage of a diffraction grating as the wavelength-selection module, tunable and single-mode emission from 310 to 363 nm was realized.

Pr^3+^ ions exhibit strong absorption at ≈450 nm, a property that perfectly matches the emission of commercially available and low-cost 450 nm blue LED chips. Consequently, it is feasible to fabricate deep UV LEDs by combining Pr^3+^-doped phosphors with a 450 nm blue chip (Figure 6c). Following this approach, Zi et al. recently developed a thumb-sized deep-UV LED by coating CaSrSiO_4_:Pr^3+^ phosphors onto a 450 nm blue LED chip.^58^ This device, powered by a button cell battery, emits light across the 250–350 nm spectral range with a central wavelength of 280 nm. Remarkably, Pr^3+^ embedded in selective hosts like LiYF_4_ and Li_2_SrGeO_4_ can produce UVC upconversion luminescence via direct excitation from sunlight.^59,60^ This capability opens a compelling avenue for future applications, namely, the construction of self-powered deep-UV emission systems.

### 4.2 Photocatalysis and Photoreaction

Pr^3+^-doped inorganic compounds capable of converting visible light into UV radiation hold significant promise in catalytic applications (Figure 6d). Our group demonstrated YOBr:Pr^3+^-activated photocatalysis for overall water splitting under visible light excitation.^2^ When irradiated by visible light from a xenon lamp (>420 nm), YOBr:Pr^3+^ phosphors emitted UV upconversion photons that can be absorbed by NiO-loaded NaTaO_3_:La inside water, eventually leading to H_2_ gas generation. Notably, the solar spectrum peaks at ≈450 nm, which coincides with the strongest upconversion excitation wavelength of Pr^3+^. Therefore, this system could potentially harness sunlight—an abundant and sustainable energy source—as the excitation light in future applications.

Many commercially available photoinitiators, such as photocurable resins and commercial photoresists (e.g., SU-8), require UV light to induce their activation and initiate the radical polymerization of photosensitive resins (Figure 6e).^61^ Therefore, UCNPs capable of converting NIR light into UV light open up unique possibilities for developing novel NIR-triggered photopolymerization technologies. As a proof of concept, we demonstrated the precise fabrication of SU-8 polymer microstructures directly on top of a microring resonator. The device was prepared by sequentially depositing NaErF_4_@NaYF_4_ UCNPs and SU-8 onto the substrate, after which the polymer structures were developed by locally generated UV light above the resonator. Significantly, this approach enabled the curing of a 2 μm-thick structure using a 1550 nm laser at a low excitation power of 20 μW within 10 min, significantly faster than conventional lithography.^4^

UVC photons demonstrate microbial inactivation owing to their spectral overlap with the absorption peaks of DNA/RNA (Figure 6f).^62^ Yang and co-workers designed and validated the bactericidal efficacy of a series of Pr^3+^-doped systems, including Sr_2_SiO_4_:Pr^3+^, Ca_2_SiO_4_:Pr^3+^, Ba_2_SiO_4_:Pr^3+^, Li_2_SrGeO_4_:Pr^3+^, SrSiO_3_:Pr^3+^, and CaSrSiO_4_:Pr^3+^.^58,59,63–65^ One key advantage of UV upconversion photons is that certain Pr^3+^-doped systems can convert sunlight into UVC photons, enabling efficient solar energy utilization. In this context, it can serve as a promising supplement to mainstream deep-UV LEDs. Another advantage is their ability to disinfect sealed glass containers, a task unachievable with conventional deep-UV LEDs since most commercially available glass materials are opaque to UVC radiation.^63^

UV upconversion materials can generate energetic photons in natural environments to promote the cleavage of plastic backbones. Chen’s group demonstrated that incorporating Li_2_CaGeO_4_:Pr^3+^ upconversion phosphors as additives dramatically accelerated plastic degradation (38-fold increase in surface cracking area) through visible-to-UV photon upconversion, pioneering a sustainable approach for addressing plastic pollution.^66^ In addition, UV UCNPs can destroy cancer cells by converting tissue-penetrating NIR light into cytotoxic UV light for biomedical applications. The UV light generates highly toxic hydroxyl radicals (•OH) by accelerating Fenton chemistry and produces reactive oxygen species (ROS) by exciting the natural photosensitizers of cells, both of which lead to cell death.^67,68^

### 4.3 Solar Blind Imaging

Building upon the absence of terrestrial UVC photons, one can exploit the unique feature of UV upconversion for advanced solar blind imaging applications such as static labeling, dynamic tracking, and information encryption. In a representative example, Zi et al. demonstrated effective localization of a moving vehicle equipped with a portable upconverted UVC-LED (Figure 7a).^59^ Remarkably, this approach maintains robust tracking performance even in the presence of minor obstructions, such as interference from branches in a navigation task through dense foliage. As the spectral peak of solar emission coincides with the excitation band of Pr^3+^ ions, this technology can even achieve its intended functionality under the excitation of sunlight without the need for additional energy input (Figure 7b).^65^

**Figure 7:**
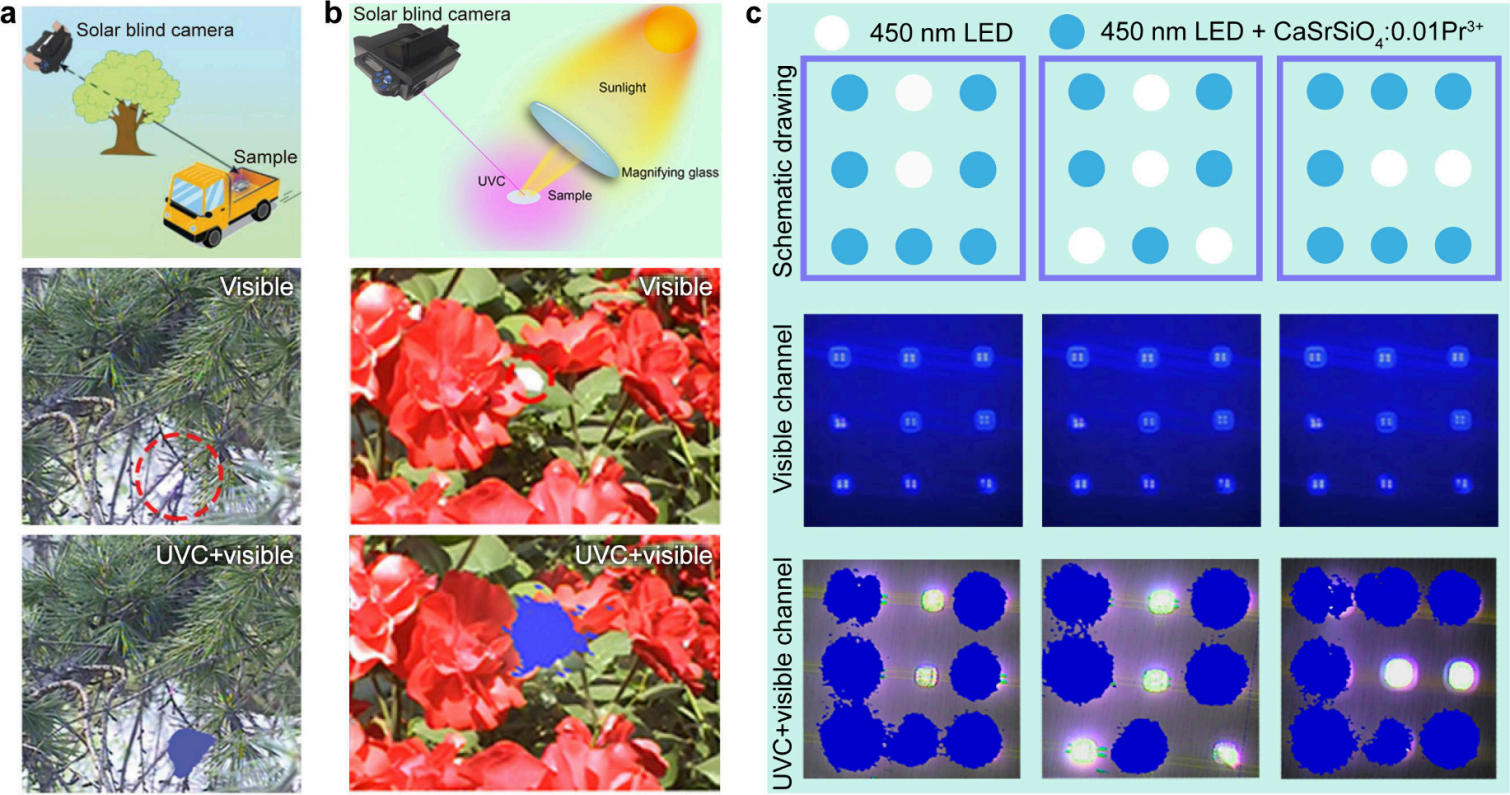
(a) Dynamic tracking of a trolley by capturing the UVC emission from Li_2_SrGeO_4_:Pr^3+^ coated on a 450 nm LED chip. Reproduced with permission from ref 59. Copyright 2024 Wiley-VCH. (b) Static labeling by capturing the UVC emission from a SrSiO_3_:Pr^3+^ ceramic excited by sunlight. Reproduced with permission from ref 65. Copyright 2023 Royal Society of Chemistry. (c) Information encryption. Reprinted with permission from ref 58. Copyright 2025 AIP Publishing

This solar-excited upconversion property also facilitates passive information encryption and anticounterfeiting, as well as dynamic tracking applications without the need for external power input. As illustrated in Figure 7c (top panel), a 3 × 3 array configuration was fabricated using nine blue LEDs, with selected units coated with Pr^3+^-doped UVC upconversion phosphor. Initial imaging through the visible light channel of the solar-blind camera revealed only the basic LED array structure, yielding no discernible encoded information (middle panel). However, upon switching to the composite detection mode incorporating both visible and UVC channels, the previously concealed information “UVC” became clearly resolvable (bottom panel).^58^

While the use of upconversion materials as NIR- or visible-excitable UVC emitters for solar-blind imaging is a promising concept, it is crucial to acknowledge several inherent challenges. A key limitation, common to all UVC-based imaging, is the shallow penetration depth of UVC photons, which confines applications primarily to surface-level analysis. Furthermore, the overall signal strength is contingent not only on the detector sensitivity but more critically on the intensity of UVC emitters. This constraint necessitates either the use of highly sensitive (and often costly) solar-blind detectors or high excitation power that raises concerns about long-term photostability.

## 5 SUMMARY AND OUTLOOK

We have reviewed the recent progress in UV upconversion, primarily drawing from our efforts in designing and engineering lanthanide-doped inorganic crystals. By strategic doping of lanthanide ions in judiciously designed host materials, such as core–shell nanoparticles, tunable UV emissions can now be readily achieved in a diversity of materials systems by visible or NIR excitation. Meanwhile, several approaches have been established to enhance the UV upconversion luminescence intensity by exploiting extrinsic factors, such as surface-capped organic molecules. These advances have significantly expanded the range of applications for upconversion in photonics and biological sciences. In particular, the Pr^3+^-based upconversion systems even demonstrate deep-UV emissions by excitation of solar radiation, which may offer a sustainable approach for managing environmental wastes.

Despite these remarkable achievements, the exploration of UV upconversion is still a developing field, with numerous challenges and exciting opportunities on the horizon. Looking forward, the prospects for UV upconversion are exceptionally promising and will likely be driven by progress in the following directions:

**(I) Expanding the toolbox of lanthanide activators for UV upconversion luminescence**. This Account focuses on Er^3+^-, Tm^3+^-, and Pr^3+^-based systems. However, a review of trivalent lanthanide energy level diagrams shows that other ions, such as Nd^3+^, also have accessible UV energy levels and thus merit future investigation. Recently, Wang et al. and Liu et al. introduced a simple yet effective approach that utilizes a broadband flashlight as the excitation source to achieve UV upconversion emission from Er^3+^, Tm^3+^, Ho^3+^, Nd^3+^, and Pr^3+^.^69,70^ The flashlight spectrum spans 400–760 nm, enabling these ions to generate UV upconversion by absorbing multiple photons of different wavelengths. This strategy contrasts with the conventional use of a single-wavelength laser for excitation, offering valuable inspiration for future research.

**(II) Boosting UV upconversion luminescence efficiency**. The practical application of UV upconversion is currently constrained by low quantum yields and the need for high-power excitation (Table 1).^39,71–74^ The intense irradiation required for multiphoton processes induces significant local heating (photothermal effects), which in turn causes thermal quenching and sharply diminishes luminescence efficiency. High-power excitation poses an additional challenge of long-term photostability as it degrades nanomaterials through surface ligand loss, aggregation, or irreversible phase transitions. Future strategies must therefore focus on enhancing the upconversion efficiency to reduce the excitation power threshold. Coupling visible-light-absorbing dyes with Pr^3+^ ions may be a promising strategy for achieving efficient UV upconversion under low-intensity excitations.

**Table 1:** Comparison of Excitation Wavelength (λ_ex_), Excitation Power Density (*P*_ex_), and Quantum Yield (Φ_UC_) of Representative UV Upconversion Materials

Material system	λ_ex_ (nm)	*P*_ex_ (W cm^–2^)	Φ_UC_ (%)	Ref.
NaGdF_4_:Yb/Tm@NaGdF_4_:Yb@NaGdF_4_:Yb/Nd@NaGdF_4_	808	NA	0.04 (240–400 nm)	^40^
NaGdF_4_:Yb/Tm@NaYF_4_:Yb@NaGdF_4_:Yb/Nd@NaGdF_4_	808	NA	0.13 (240–400 nm)	^40^
SrYbF_5_@SrYbF_5_:Tm@SrYbF_5_@SrYF_5_	980	80	0.13 (350–400 nm)	^71^
LiYbF_4_:Tm@LiYF_4_	980	100	0.15 (330–400 nm)	^72^
LiYbF_4_:Tm@LiYF_4_/CsPbCl_3_	980	100	0.08 (330–380 nm)	^72^
Rb_3_InCl_6_:Yb/Er (nanocrystals)	980	60	0.08 (∼384 nm)	^73^
Rb_3_InCl_6_:Yb/Er (microcrystals)	980	60	0.12 (∼384 nm)	^73^
Cs_2_NaYCl_6_:Pr	450.9	0.59	0.11 (250–340 nm)	^74^
LuAG:Pr	450.9	0.59	0.025 (250–340 nm)	^74^
YAG:Pr	450.9	0.59	0.009 (250–340 nm)	^74^

**(III) Establishing theory framework for structure–function relationship**. A pivotal future direction for UV upconversion research is the shift from trial-and-error discovery toward a theory-guided rational design framework. Establishing a robust understanding of structure–property relationships is key to streamlining materials development. Computational tools are central to this effort. First-principles calculations (e.g., DFT) provide a theory-driven pathway for screening potential host materials by assessing their intrinsic electronic and phonon properties, thereby filtering out candidates susceptible to nonradiative losses. In parallel, data-driven machine learning can analyze extensive experimental data to identify hidden correlations, enabling an “inverse design” approach where optimal material compositions can be predicted from a targeted performance metrics.
